# Integration Profiling Between Plasma Lipidomics, Epstein–Barr Virus and Clinical Phenomes in Nasopharyngeal Carcinoma Patients

**DOI:** 10.3389/fmicb.2022.919496

**Published:** 2022-06-30

**Authors:** Yi Huang, Jinfeng Liang, Wenjin Hu, Yushan Liang, Xue Xiao, Weilin Zhao, Xuemin Zhong, Yanping Yang, Xinli Pan, Xiaoying Zhou, Zhe Zhang, Yonglin Cai

**Affiliations:** ^1^Department of Otolaryngology-Head and Neck Surgery, First Affiliated Hospital of Guangxi Medical University, Nanning, China; ^2^State Key Laboratory of Non-Food Biomass and Enzyme Technology, Guangxi Key Laboratory of Bio-refinery, National Engineering Research Center for Non-Food Biorefinery, Guangxi Biomass Engineering Technology Research Center, Guangxi Academy of Sciences, Nanning, China; ^3^Key Laboratory of Early Prevention and Treatment for Regional High-Frequency Tumor, Guangxi Key Laboratory of High-Incidence-Tumor Prevention and Treatment, Ministry of Education, Guangxi Medical University, Nanning, China; ^4^Guangxi Key Laboratory of Marine Natural Products and Combinatorial Biosynthesis Chemistry, Beibu Gulf Marine Research Center, Guangxi Academy of Sciences, Nanning, China; ^5^Guangxi Health Commission Key Laboratory of Molecular Epidemiology of Nasopharyngeal Carcinoma, Wuzhou Red Cross Hospital, Wuzhou, China

**Keywords:** nasopharyngeal carcinoma, Epstein–Barr virus, lipidomics, LC–MS/MS, cancer stage

## Abstract

Plasma lipidomics has been commonly used for biomarker discovery. Studies in cancer have suggested a significant alteration of circulating metabolite profiles which is correlated with cancer characteristics and treatment outcome. However, the lipidomics characteristics of nasopharyngeal carcinoma (NPC) have rarely been studied. We previously described the phenomenon of lipid droplet accumulation in NPC cells and showed that such accumulation could be regulated by latent infection of Epstein–Barr virus (EBV). Here, we compared the plasma lipidome of NPC patients to that of healthy controls by liquid chromatography-tandem mass spectrometry (LC–MS/MS). We found 19 lipids (e.g., phosphatidylinositols 18:0/20:4 and 18:0/18:2 and free fatty acid 22:6) to be remarkably decreased, whereas 2 lipids (i.e., diacylglycerols 16:0/16:1 and 16:0/20:3) to be increased, in the plasma of NPC patients, compared with controls. Different lipid profiles were also observed between patients with different titers of EBV antibodies (e.g., EA-IgA and VCA-IgA) as well as between patients with and without lymph node or distant organ metastasis. In conclusion, plasma lipidomics might help to differentiate NPC cases from controls, whereas EBV infection might influence the risk and prognosis of NPC through modulating lipid metabolism in both tumor cells and peripheral blood.

## Introduction

As reported in GLOBOCAN 2020, there were 133,354 newly diagnosed cases of nasopharyngeal carcinoma (NPC) in 2020, accounting for 0.7% of the total human cancer globally (Sung et al., 2021). NPC is prevalent in Southeast Asia, especially China, and relatively rare elsewhere (Yu and Yuan, 2002; Zhang et al., 2015; Chen et al., 2019). Multiple factors contribute to the initiation and development of NPC, among which, the crosstalk of Epstein–Barr virus (EBV), genetic factors, and environmental carcinogens likely constitutes the main contributor.

Epstein–Barr virus infection is the most established risk factor for NPC, especially for non-keratinizing NPC: the most common histological type of NPC in endemic regions (Pathmanathan et al., 1995; Yu and Hussain, 2009). EBV infection may exhibit a latent or lytic phase (Hoch et al., 1988; Moss et al., 2001). In the latent phase, the EBV genomic DNA exists like host chromosomal DNA. Expression of several EBV proteins facilitates this phase, including latent membrane proteins (LMP1, LMP2A, and LMP2B) and EBV nuclear antigens (EBNA1, EBNA2, EBNA3A, EBNA3B, and EBNA3C). The expression of the immediate-early proteins, Zta and Rta (encoded by BZLF1 and BRLF1, respectively), initiates on the other hand the lytic phase of EBV infection (Robertson and Kieff, 1995; Binné et al., 2002; Hui and Chiang, 2010). EBV reactivation may be identified by detection of IgA antibody to EBV early antigen (EA) and late antigen (VCA; Hoch et al., 1988). As early as 37 months before the clinical onset of NPC, an increased serological level of EBV VCA-IgA might already be detected (Ji et al., 2007). To date, serological detection of EBV-related antibodies including VCA-IgA, EA-IgA, and EBV nuclear antigen 1 (EBNA1)-IgA antibodies has been widely applied in NPC screening programs (Zeng et al., 1982; Liu et al., 2012; Coghill et al., 2014; Gao et al., 2017).

Accumulating evidence suggests the involvement of lipid metabolism reprogramming in cancer (Luo et al., 2017). Cancer cells increase *de novo* synthesis to better utilize lipids in energy metabolism to facilitate malignant behaviors like cell death resistance and replicative immortality enablement (Carracedo et al., 2013). In a previous study, we showed that there was an aberrant accumulation of lipid droplets in NPC cells, likely attributable to the epigenetic downregulation of the ISG15-conjugating enzyme UbcH8, leading to altered stabilization of adipose triglyceride lipase (Zhou et al., 2015). High-density lipoprotein cholesterol has also been shown to enhance proliferation, migration, colony formation, and chemoresistance ability of NPC cells, whereas a higher level of high-density lipoprotein cholesterol was found to predict a poor survival rate of NPC patients (Liu et al., 2016).

It is known that there is an interplay between host cell lipid metabolism and infection of oncogenic viruses including EBV (Daker et al., 2013). EBV infection may therefore promote the development of NPC through modulating lipid metabolism. EBV-encoded latent membrane protein 1 may promote the proliferation and progression of NPC by activating the sterol regulatory element-binding protein 1 and its downstream molecular fatty acid synthase, leading to an increased *de novo* lipid synthesis and droplet formation (Lo et al., 2018). Our previous study also showed that EBV-encoded latent membrane protein 2A (LMP2A) could drive the downregulation of adipose triglyceride lipase, leading to lipid accumulation and migration of NPC cells (Zheng et al., 2020). EBV-encoded RNAs are found highly expressed in EBV-positive NPC cells (Raab-Traub, 2015). Although they do not seem to affect the migration or invasion of cancer cells, EBV-encoded RNAs might confer a low-density lipoprotein-dependent proliferation in NPC cells (Daker et al., 2013). Furthermore, EBV could target adipocytes to build a cancer-promoting environment by rewiring lipid metabolism, leading to increased levels of free fatty acids, glycerol, and proinflammatory cytokines (Liu et al., 2021). Indeed, increased serum levels of free fatty acids have been shown to indicate a poor prognosis in NPC patients (Liu et al., 2021).

Lipidomics identifies changes in lipid metabolism, transport and homeostasis and has played an important role in understanding biochemical mechanisms of lipid-related diseases (Han, 2016). Recent advances in mass spectrometry and other spectroscopic methods have further promoted the development and use of lipidomics in research (Hu et al., 2009). For instance, liquid chromatography-tandem mass spectrometry (LC–MS/MS) has been used widely in studying complex metabolomes (Leung and Fong, 2014). In terms of cancer, lipidomics has been used in studies of lung cancer (Zhang et al., 2020), bladder cancer (Shahid et al., 2020), esophageal cancer (Liu et al., 2013), colorectal cancer (Tan et al., 2013), thyroid cancer (Jiang et al., 2021), and breast cancer (Fichtali et al., 2020). However, to our best knowledge, no study has examined lipidomics in NPC, to date.

To this end, we used LC–MS/MS to determine the lipid profiles in the plasma of patients with NPC, through comparing different lipid metabolites between NPC patients and healthy controls in an endemic area of Southeast China. We also studied the correlations between lipid profiles with EBV infection, measured through EBV antibodies, and clinical characteristics of NPC, namely TMN and clinical stages.

## Materials and Methods

### Study Design

We recruited 100 NPC patients and 100 age- and sex-matched healthy controls in Wuzhou Red Cross Hospital (Wuzhou, China) between 2018 and 2020. The diagnosis of NPC was cross validated by two experienced pathologists according to the World Health Organization classification (Stelow and Wenig, 2017). The patients were required to have not received radiation- or chemotherapy and have no hyperlipidemia, diabetes, or other conditions affecting lipid metabolism. Patients with other malignancy or recurrent NPC were also excluded. The healthy controls were required to have no metabolic disorders and no EBV VCA-IgA and EA-IgA antibodies. We collected information on clinical characteristics of the NPC patients, including age, sex, EBV antibodies (VCA-IgA, EA-IgA, EBNA1-IgA, and Zta-IgA), as well as Tumor Node Metastasis (TNM) stage and clinical stage defined according to the eighth edition of the American Joint Committee on Cancer (AJCC) staging system.

Ethics approval of this study was obtained from the Ethical Evaluation Committee of Wuzhou Red Cross Hospital (No. LL2017-19, Wuzhou, China). All participants signed informed consent before recruitment to the study.

### EBV Antibody Detection in Serum

A morning fasting peripheral blood (5 ml) sample was collected using a non-anticoagulant-treated tube. The blood samples were allowed to clot at room temperature for half an hour. The samples were then centrifuged at 2,000 rmp at 4°C for 10 min, with the supernatant serum transferred, aliquoted, and stored at –70°C for further analysis of the serological levels of VCA-IgA and EA-IgA antibodies using an immunoenzymatic assay as previously described (Cai et al., 2014). All controls had negative results on these antibodies, whereas NPC patients were classified as with high or low titers of these antibodies based on the median titers of the antibodies among all patients with NPC. Information on EBV Zta-IgA and EBNA-IgA antibodies (classified as negative or positive) was obtained from medical records review and only available for NPC patients. EBNA1-IgA and Zta-IgA antibodies were measured in Wuzhou Red Cross Hospital using commercial kits (Zhongshan Biotech, Zhongshan, China) based on enzyme-linked immunosorbent assay (ELISA).

### Lipid Extraction and LC–MS/MS

We collected another sample of remaining morning fasting blood in the anticoagulant-treated tubes at 2,000 rmp at 4°C for 10 min. The supernatant plasma was transferred, aliquoted, and stored at −80°C before the samples were sent to the Shanghai Institute of Clinical Bioinformatics where 20 μl plasma was added into a glass tube to extract the total lipids using the isopropanol precipitation method (Sarafian et al., 2014). Briefly, 9 μl internal standard cocktails (AB SCIEX, 5040146-5,040,155) and 350 μl pre-cold isopropanol were added to each tube which was then shaken vigorously and incubated at −20°C overnight. On the next day, samples were centrifuged at 14,000 rpm at 4°C for 20 min with the supernatant transferred and centrifuged again under the same condition. Finally, the supernatant was ready for lipid extraction.

All samples were measured in the normal-phase liquid chromatography-coupled triple-quadrupole mass spectrometers (Q-trap^®^ 5,500 LC–MS/MS, Sciex, Framingham, MA, United States of America). The determination conditions used in LC–MS/MS were as follows: Acquity UPLC BEH HILIC (Waters Co., Milford, MA, US) column (100 mm × 2.1 mm, 1.7 μm) was used as stationary phase, acetonitrile solution (containing 10 mmol/l ammonium acetate) was used as mobile phase A, and 50% acetonitrile solution (containing 10 mmol/l ammonium acetate) was used as mobile phase B. The gradient elution of mobile phase B: 0–10 min, raised from 0.1 to 20%; 10–11 min, raised from 20 to 98%; 11–13 min, maintained at 98%; 13.1 min, reduced to 0.1%; and 13.2–16 min, maintained at 0.1%. Other conditions mainly included a flow rate of 0.5 ml/min, a column temperature of 35°C, an injection volume of positive/negative ion mode at 4 μl, a gas curtain pressure at 35 psi, a nebulizer pressure at 50 psi, an auxiliary gas pressure at 60 psi, and a heating temperature at 500°C. The positive and negative ion mode ion spray voltages were − 5,500 V and 5,500 V, the positive and negative ion mode optimized de-clustering voltages were 80 V and − 80 V, whereas the positive and negative injection voltages were 10 V and − 10 V, respectively.

An equal amount of liquid harvested from each sample was mixed and used as a quality control (QC) mixture. The QC sample was treated the same as the study samples. One QC sample was added to each ten study samples. The stability of LC–MS/MS was evaluated by principal component analysis of QC samples and other experimental samples. On the principal component diagram, the projection points of QC samples were relatively clustered, demonstrating that LC–MS/MS had good stability and repeatability. The results also showed that the differences among the test samples were mainly due to the variations in the metabolites of the test samples instead of the variations of the test methods. The production was scanned by Q-Trap in multiple reaction monitoring modes.

### Statistical Analysis

Among all detected lipids, we retained the ones with > 30% of non-zero values among either the cases or controls and disregarded the rest. After this data filtering, we retained 1,125 of the total 1,191 lipid molecules detected in LC–MS/MS. Using the filtered data, we performed a case–control comparison as well as subgroup analyses of the NPC patients by EBV antibodies, tumor stage, and sex. To address whether the sex-specific lipid profiles were specific to NPC, we also compared the lipids between male and female controls. In the subgroup analyses, if a lipid molecule had < 5% non-zero values in either of the comparison groups, we discarded such comparison. We used unpaired Welch *t* test in all comparisons, considering the homogeneity of variance of the samples. Both fold-change value and *p* value were calculated in these analyses. A fold-change value was computed for each lipid molecule through comparing the averages of different groups. A *p* value <0.05 was considered statistically significant. Volcano plots were used to present the lipid molecules that differed statistically significantly between groups (with a fold-change > 2 and a *p* value < 0.05).

We also performed multivariable analyses, namely partial least squares discriminant analysis (PLS-DA) and orthogonal partial least squares discriminant analysis (OPLS-DA) to examine whether the lipid profiles can be used to discriminate NPC patients from healthy controls. PLS-DA and OPLS-DA are supervised methods to establish a model of the relationship between the predictor (i.e., a lipid profile) and outcome (i.e., case–control status) and to subsequently estimate the usefulness of the predictor (Worley and Powers, 2013). Parameters *R*^2^ and *Q*^2^ were used to evaluate the interpretability and predictability of the PLS-DA and OPLS-DA models, respectively. A VIP value was used to indicate the contribution of the predictor to the OPLS-DA model to discriminate cases from healthy controls. The greater the contribution, the higher the VIP value. We used a 7-fold cross-validation and permutation test with 200 repeats when constructing the PLS-DA model. R version 4.0.5 was used in all data analyses and for graphic visualization.

## Results

Table 1 shows the characteristics of the NPC patients and their age- and sex-matched controls. There were 27 female and 73 male among the NPC patients, with a mean age of 49.4 years (range: 23–87 years), and 27 female and 73 male among the controls, with a mean age of 48.6 years (range: 17–91 years).

**Table 1:** Characteristics of nasopharyngeal carcinoma (NPC) patients and their age- and sex-matched healthy controls.

Characteristics	Control group (*N* = 100)	NPC group (*N* = 100)
Age (years)		
Mean ± SD	48.6 ± 20.4	49.4 ± 10.8
Range	17–91	23–87
Sex		
Female	27	27
Male	73	73
T stage		
T1		3
T2		30
T3		31
T4		36
N stage		
N0		7
N1		22
N2		40
N3		31
M stage		
M0		91
M1		8
Mx		1
Clinical stage		
II		10
III		32
IVA		49
IVB		9
EBV Zta-IgA	Not tested	
Positive		34
Negative		44
Not test		22
EBNA1-IgA	Not tested	
Positive		66
Negative		12
Not test		22
EBV VCA-IgA (titers)^*^	Negative	
≤1:320		47
>1:320		28
0 Not test		3 22
EBV EA-IgA (titers)^*^	Negative	
≤ 1:80		42
**>** 1:80 0 Not test		29 8 22

* Classification was based on median titer of the antibodies.

The numbers of NPC patients do not add up to 100 because some patients were negative for the antibodies (3 for VCA-IgA and 8 for EA-IgA) whereas some were not measured.

We identified 1,191 lipids in both the NPC patients and controls, including 3 cholesterols, 21 cholesterol esters, 2 ceramides, 12 sphingomyelins, 1 sphingosine, 1 sphingosine-1-phosphate, 17 monoacylglycerols, 50 diacylglycerols, 445 triglycerides, 77 phosphatic acids, 141 phosphatidylethanolamines, 77 phosphatidylglycerols, 76 phosphatidylinositols, 77 phosphatidylserines, 78 phosphatidylcholine, 13 lyso-phosphatic acids, 16 lyso-phosphatidylethanolamines, 16 lyso-phosphatidylglycerols, 16 lyso-phosphatidylinositols, 16 lyso-phosphatidylserines, 16 lyso-phosphatidylcholine and 20 free fatty acids. After data filtering, we retained 1,125 of these lipids in the analysis.

### Lipid Profiles Between NPC Patients and Controls

**Figure 1:**
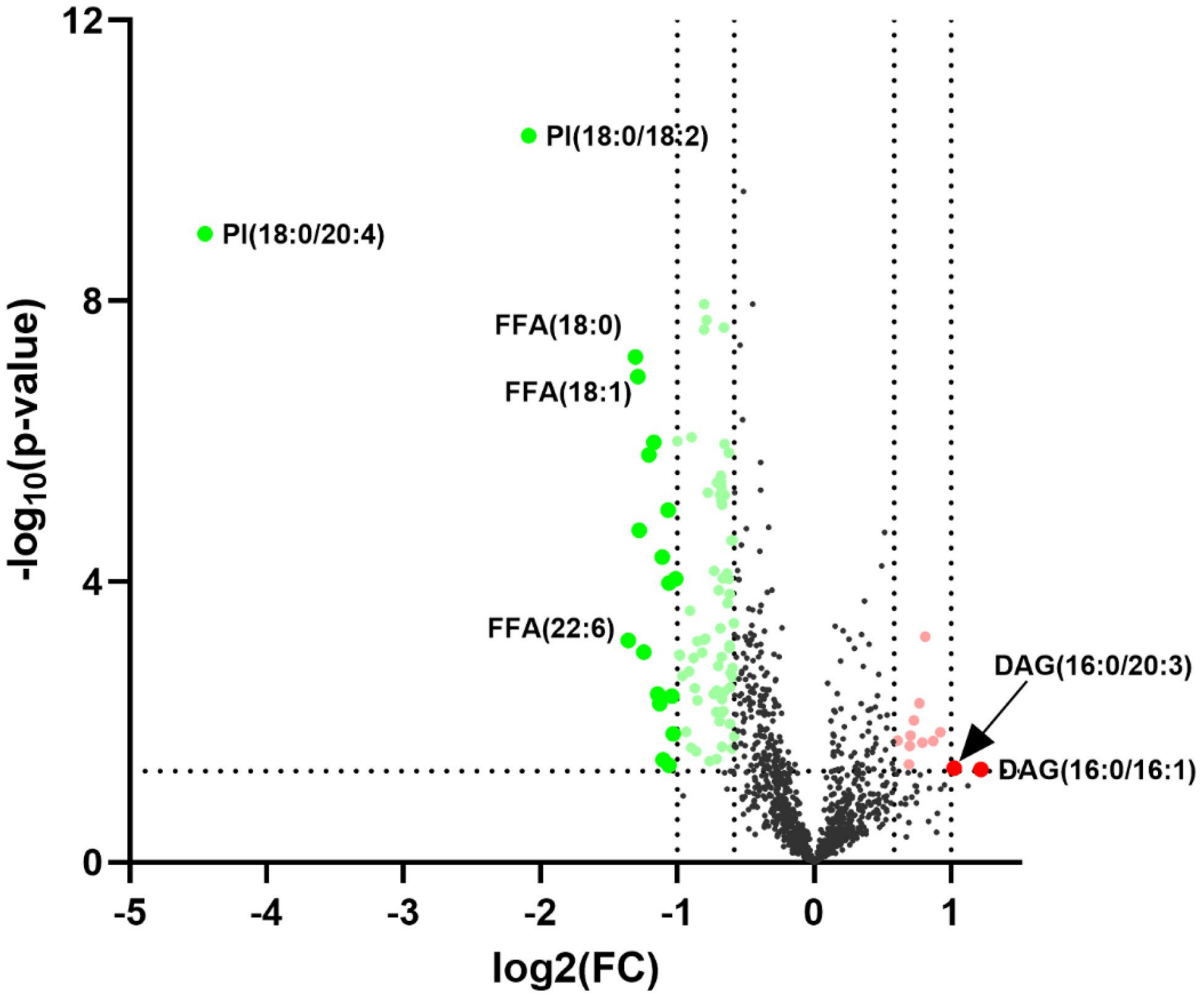
Volcano plot for the 21 lipid molecules showing statistically significantly different levels between nasopharyngeal carcinoma (NPC) patients and controls. The *x*-axis shows Log_2_ (FC), the *y*-axis shows –Log_10_ (*p* value) and each dot represents one lipid. The big red and green dots above the straight line [−Log_10_(0.05)] show lipids with statistically significantly different levels between NPC patients and controls as well as a fold-change (FC) greater than 2 (alternatively smaller than 0.5). PI, phosphatidylinositol; FFA, free fatty acid; DAG, diacylglycerol.

**Figure 2:**
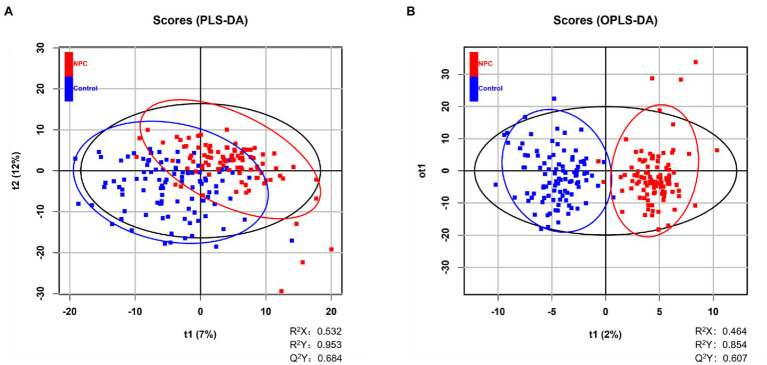
Distinguishing NPC patients from controls using supervised statistical analyses. **(A)** NPC patients and controls are partially distinguished based on 2-dimensional scores made by PLS-DA, where *Q*^2^ > 0.5 and *R*^2^ > 0.5 indicate the predictive ability of the model. A blue square represents a healthy control whereas a red square represents an NPC patient. **(B)** Score plot of OPLS-DA showing the separation of NPC patients and controls, where *Q*^2^ > 0.5 and *R*^2^ > 0.5 indicate the predictive ability of the model. A blue square represents a healthy control whereas a red square represents an NPC patient.

**Figure 3:**
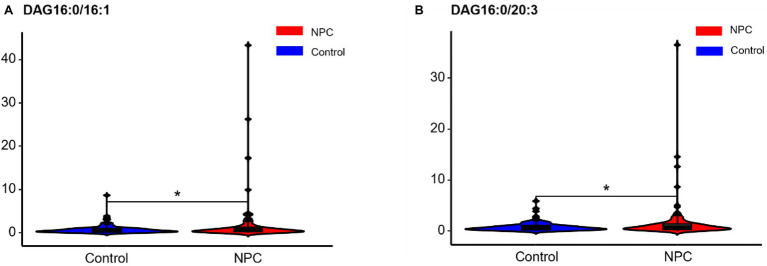
Violin plots showing the two lipids with a higher level among NPC patients compared with controls. ^*^ means *p* < 0.05. NPC means nasopharyngeal carcinoma. Control means healthy controls. DAG, diacylglycerol.

**Figure 4:**
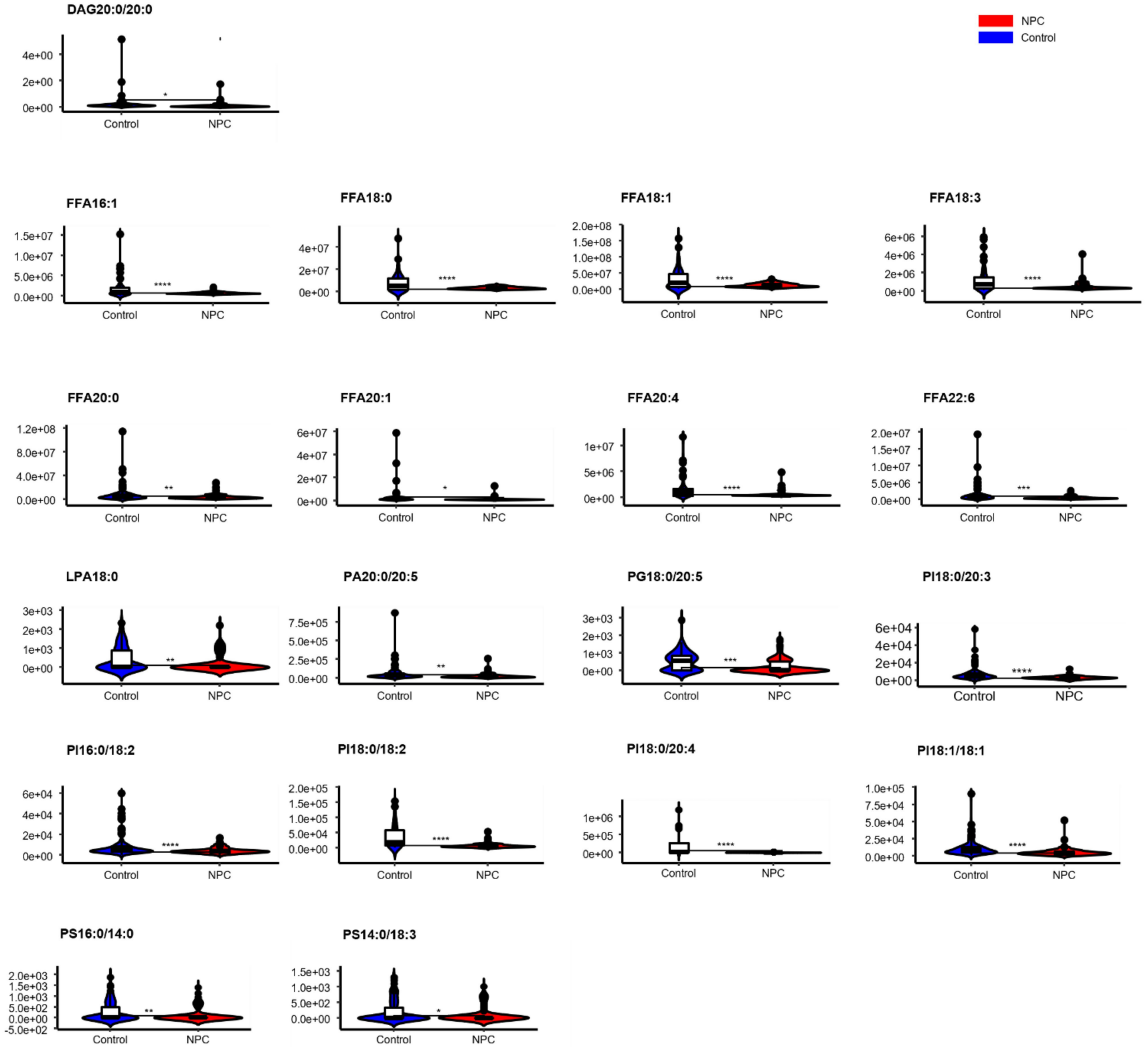
Violin plots showing the 19 lipids with a lower level among NPC patients compared with controls. ^*^ means *p* < 0.05, ^**^ means *p* < 0.01, ^***^ means *p* < 0.001, ^****^ means *p* < 0.0001, and “ns” means *p* > 0.05. DAG, diacylglycerol; FFA, free fatty acid; PI, phosphatidylinositol; PG, phosphatidylglycerol; PS, phosphatidylserine; PA, phosphatidic acid; LPA, lyso- phosphatidic acid.

Statistically significant differences were noted for 349 lipids between NPC patients and controls (*p* value < 0.05). Figure 1 shows the lipid molecules with a fold-change > 2 (alternatively < 0.5) of these 349 lipids, when comparing NPC patients to controls. Figure 2A shows the results of PLS-DA model and Figure 2B shows the results of OPLS-DA model. The *R*^2^ and *Q*^2^ values of the score plots suggest that the models are stable with good predictive ability. A statistically significant difference was noted for 21 lipid molecules between NPC patients and controls, including three classes, namely free fatty acids, glycerolipids, and glycerophospholipids, as well as seven sub-classes (Table 2). Among these, diacylglycerols 16:0/20:3 and 16:0/16:1 showed a higher level (Figure 3), whereas the other 19 lipids, including diacylglycerol 20:0/20:0, free fatty acids 16:1, 18:0, 18:1, 18:3, 20:0, 20:1, 20:4, and 22:6, lyso-phosphatic acid 18:0, phosphatic acid 20:0/20:5, phosphatidylglycerol 18:0/20:5, phosphatidylinositols 16:0/18:2, 18:0/18:2, 18:0/20:3, 18:0/20:4, and 18:1/18:1, as well as phosphatidylserines 14:0/18:3 and 16:0/14:0 showed a lower level (Figure 4), among NPC patients than controls.

**Table 2:** Lipids with different levels between patients with NPC and controls.

No.	Lipid	Class	*p* Value^a^	FC^b^	Log_2_(FC)	VIP value^c^
1	DAG16:0/16:1	Glycerolipids	0.047	2.325	1.217	1.495
2	DAG16:0/20:3	Glycerolipids	0.046	2.028	1.02	1.447
3	PI16:0/18:2	Glycerophospholipids	9.18E-05	0.479	−1.011	2.289
4	PS14:0/18:3	Glycerophospholipids	0.015	0.463	−1.03	1.179
5	FFA20:0	Fatty acids	0.004	0.404	−1.041	1.359
6	PG18:0/20:5	Glycerophospholipids	0.0001	0.409	−1.062	1.741
7	DAG20:0/20:0	Glycerolipids	0.041	0.433	−1.062	1.215
8	PI18:1/18:1	Glycerophospholipids	9.62E-06	0.486	−1.069	2.137
9	FFA20:1	Fatty acids	0.035	0.465	−1.106	1.152
10	FFA16:1	Fatty acids	4.47E-05	0.412	−1.111	1.810
11	PS16:0/14:0	Glycerophospholipids	0.005	0.389	−1.129	1.095
12	PA20:0/20:5	Glycerophospholipids	0.004	0.421	−1.145	1.308
13	PI18:0/20:3	Glycerophospholipids	1.04E-06	0.452	−1.171	2.815
14	FFA18:3	Fatty acids	1.57E-06	0.479	−1.209	2.649
15	LPA18:0	Glycerophospholipids	0.001	0.496	−1.246	1.396
16	FFA20:4	Fatty acids	1.85E-05	0.235	−1.278	2.340
17	FFA18:1	Fatty acids	1.19E-07	0.444	−1.288	2.870
18	FFA18:0	Fatty acids	6.27E-08	0.046	−1.307	2.888
19	FFA22:6	Fatty acids	0.00069	0.477	−1.359	1.679
20	PI18:0/18:2	Glycerophospholipids	4.40E-11	0.489	−2.087	3.472
21	PI18:0/20:4	Glycerophospholipids	1.10E-09	0.457	−4.449	3.097

a *p* value was calculated using unpaired *t* test.

b Fold-change (FC) value was calculated by comparing the averages of NPC patients to that of the healthy controls.

c VIp value was obtained from OPLS-DA models.

### Lipid Profiles in NPC Patients by EBV Antibodies

There was a lower level of one lipid (phosphatidic acid 14:0/20:1) whereas a higher level of 19 lipids, including 18 glycerolipids, among NPC patients with a lower titer of EA-IgA antibodies, compared with NPC patients with a higher titer of EA-IgA antibodies (Table 3). There was a higher level of 52 lipids, all glycerolipids, among NPC patients with a lower titer of VCA-IgA antibodies, compared with patients with a higher titer of VCA-IgA (Table 4). NPC patients with the presence of Zta-IgA had a higher level of 2 lipids (diacylglycerol16:1/20:2 and triacylglycerol54:0-FA16:0), whereas a lower level of 1 lipid (phosphatidic acid 16:0/14:0), compared with NPC patients without Zta-IgA. Finally, NPC patients with the presence of EBNA1-IgA had a lower level of 2 lipids whereas a higher level of 143 lipids, compared with patients without EBNA1-IgA. Table 5 shows the top 20 lipids of these.

**Table 3:** Lipids with different levels by EBV EA-IgA titers among NPC patients.

No.	Lipid	Class	*p* Value^a^	FC^b^	Log_2_(FC)
1	PA14:0/20:1	Glycerophospholipids	0.049	0.475	−1.073
2	PI16:0/20:5	Glycerophospholipids	0.013	2.703	1.435
3	DAG14:0/18:3	Glycerolipids	0.021	4.488	2.166
4	DAG18:2/18:3	Glycerolipids	0.043	2.485	1.313
5	TAG42:1-FA16:0	Glycerolipids	0.041	3.468	1.794
6	TAG42:1-FA16:1	Glycerolipids	0.042	3.349	1.744
7	TAG42:2-FA18:2	Glycerolipids	0.049	3.048	1.608
8	TAG44:2-FA16:0	Glycerolipids	0.026	2.92	1.546
9	TAG44:3-FA18:2	Glycerolipids	0.031	2.333	1.222
10	TAG46:2-FA16:1	Glycerolipids	0.039	2.061	1.043
11	TAG47:0-FA17:0	Glycerolipids	0.042	2.637	1.399
12	TAG48:4-FA16:0	Glycerolipids	0.034	2.065	1.046
13	TAG49:0-FA16:0	Glycerolipids	0.037	2.061	1.044
14	TAG49:1-FA16:1	Glycerolipids	0.023	2.088	1.062
15	TAG50:5-FA16:0	Glycerolipids	0.014	2.201	1.138
16	TAG50:5-FA20:5	Glycerolipids	0.029	2.48	1.310
17	TAG51:4-FA20:4	Glycerolipids	0.021	2.099	1.070
18	TAG52:5-FA20:5	Glycerolipids	0.045	2.207	1.142
19	TAG52:6-FA20:5	Glycerolipids	0.03	2.177	1.122
20	TAG53:4-FA20:4	Glycerolipids	0.045	2.066	1.047

a *p* value was calculated using unpaired *t* test.

b Fold-change (FC) value was calculated by comparing the averages of NPC patients with an EBV EA-IgA titer ≤ 1:80 to that of patients with an EBV EA-IgA titer > 1:80.

**Table 4:** Lipids with different levels by EBV VCA-IgA titers among NPC patients.

No.	Lipid	Class	*p* Value^a^	FC^b^	Log_2_(FC)
1	DAG14:0/18:3	Glycerolipids	0.015	4.275	2.096
2	TAG42:1-FA16:1	Glycerolipids	0.023	3.515	1.814
3	TAG42:1-FA16:0	Glycerolipids	0.025	3.397	1.764
4	TAG42:0-FA16:0	Glycerolipids	0.040	3.146	1.654
5	TAG40:0-FA16:0	Glycerolipids	0.039	3.021	1.595
6	TAG42:2-FA18:2	Glycerolipids	0.031	3.007	1.588
7	TAG44:2-FA18:1	Glycerolipids	0.046	2.921	1.547
8	TAG47:0-FA17:0	Glycerolipids	0.025	2.909	1.541
9	TAG44:2-FA16:0	Glycerolipids	0.015	2.896	1.534
10	DAG16:0/18:3	Glycerolipids	0.036	2.784	1.477
11	TAG44:0-FA14:0	Glycerolipids	0.045	2.724	1.446
12	DAG16:1/18:3	Glycerolipids	0.048	2.699	1.433
13	TAG46:0-FA16:0	Glycerolipids	0.037	2.602	1.380
14	PI16:0/20:5	Glycerophospholipids	0.011	2.584	1.369
15	TAG44:1-FA16:1	Glycerolipids	0.039	2.564	1.358
16	TAG46:0-FA14:0	Glycerolipids	0.035	2.557	1.355
17	TAG46:1-FA16:1	Glycerolipids	0.037	2.505	1.325
18	TAG47:0-FA14:0	Glycerolipids	0.038	2.493	1.318
19	TAG44:1-FA16:0	Glycerolipids	0.044	2.487	1.315
20	TAG46:1-FA16:0	Glycerolipids	0.042	2.387	1.255
21	DAG18:2/18:3	Glycerolipids	0.032	2.354	1.235
22	TAG50:5-FA20:5	Glycerolipids	0.025	2.349	1.232
23	TAG44:3-FA18:2	Glycerolipids	0.021	2.258	1.175
24	TAG48:0-FA18:0	Glycerolipids	0.040	2.242	1.165
25	TAG49:0-FA16:0	Glycerolipids	0.023	2.224	1.153
26	TAG46:2-FA16:1	Glycerolipids	0.026	2.219	1.150
27	TAG48:3-FA18:3	Glycerolipids	0.025	2.217	1.149
28	TAG49:1-FA16:1	Glycerolipids	0.017	2.207	1.142
29	TAG48:1-FA16:1	Glycerolipids	0.025	2.203	1.139
30	TAG50:4-FA20:4	Glycerolipids	0.045	2.172	1.119
31	TAG47:1-FA16:1	Glycerolipids	0.049	2.166	1.115
32	TAG52:6-FA20:5	Glycerolipids	0.020	2.156	1.109
33	TAG50:5-FA16:0	Glycerolipids	0.007	2.152	1.106
34	TAG48:4-FA16:0	Glycerolipids	0.016	2.150	1.104
35	TAG52:5-FA20:5	Glycerolipids	0.032	2.147	1.102
36	TAG44:2-FA16:1	Glycerolipids	0.045	2.144	1.100
37	TAG51:4-FA20:4	Glycerolipids	0.013	2.139	1.097
38	CE20:0	Cholesterol Ester	0.027	2.133	1.093
39	TAG49:0-FA17:0	Glycerolipids	0.036	2.109	1.076
40	TAG46:2-FA16:0	Glycerolipids	0.030	2.104	1.073
41	TAG47:1-FA16:0	Glycerolipids	0.049	2.098	1.069
42	TAG48:0-FA16:0	Glycerolipids	0.024	2.096	1.068
43	TAG47:2-FA16:1	Glycerolipids	0.031	2.090	1.064
44	TAG49:1-FA17:0	Glycerolipids	0.028	2.071	1.050
45	TAG52:5-FA20:4	Glycerolipids	0.013	2.066	1.047
46	TAG52:4-FA22:4	Glycerolipids	0.039	2.054	1.038
47	TAG52:7-FA16:0	Glycerolipids	0.036	2.040	1.029
48	TAG52:1-FA20:0	Glycerolipids	0.012	2.039	1.028
49	TAG53:4-FA20:4	Glycerolipids	0.032	2.036	1.026
50	TAG50:5-FA20:4	Glycerolipids	0.041	2.029	1.021
51	TAG52:4-FA20:4	Glycerolipids	0.019	2.015	1.011
52	TAG51:0-FA16:0	Glycerolipids	0.029	2.013	1.009

a *p* value was calculated using unpaired *t* test.

b Fold-change (FC) value was calculated by comparing the averages of NPC patients with an EBV VCA-IgA titer ≤ 1:320 to that of patients with an EBV VCA-IgA titer > 1:320.

**Table 5:** Top 20 lipids with different levels by presence of EBNA1-IgA among NPC patients.

No.	Lipid	Class	*p* Value^a^	FC^b^	Log_2_(FC)
1	PA18:0/14:0	GPs^c^	0.0232	0.4663	−1.1005
2	PS14:0/22:6	GPs^c^	0.0064	0.4389	−1.1878
3	DAG14:0/20:4	Glycerolipids	0.0046	6.7736	2.7599
4	TAG42:0-FA14:0	Glycerolipids	0.0032	6.3086	2.6573
5	TAG44:0-FA14:0	Glycerolipids	0.0022	5.7666	2.5277
6	TAG45:0-FA14:0	Glycerolipids	0.0007	5.7455	2.5224
7	TAG44:1-FA14:0	Glycerolipids	0.0006	5.7015	2.5113
8	TAG48:4-FA20:4	Glycerolipids	0.0006	5.6532	2.4991
9	TAG42:1-FA16:1	Glycerolipids	0.0035	5.5706	2.4778
10	TAG44:0-FA16:0	Glycerolipids	0.0027	5.4392	2.4434
11	TAG42:1-FA14:0	Glycerolipids	0.0031	5.3148	2.4100
12	TAG40:0-FA16:0	Glycerolipids	0.0061	4.5699	2.1922
13	TAG45:1-FA18:1	Glycerolipids	0.0290	4.5493	2.1856
14	TAG47:0-FA14:0	Glycerolipids	0.0017	4.5018	2.1705
15	TAG42:1-FA18:1	Glycerolipids	0.0029	4.2638	2.0921
16	TAG42:1-FA16:0	Glycerolipids	0.0088	4.1094	2.0389
17	TAG42:0-FA16:0	Glycerolipids	0.0103	4.0945	2.0337
18	TAG44:2-FA14:0	Glycerolipids	0.0006	4.0759	2.0271
19	TAG47:0-FA17:0	Glycerolipids	0.0055	4.0395	2.0142
20	TAG46:0-FA18:0	Glycerolipids	0.0017	3.9096	1.9670

a *p* value was calculated using unpaired *t* test.

b Fold-change (FC) value was calculated by comparing the averages of NPC patients with the presence of EBNA1-IgA antibodies to that of patients without such.

c GP, glycerophospholipids.

### Lipid Profiles in NPC Patients by Tumor Stage

No different levels of lipids were found when comparing NPC patients with a T1 or T2 tumor to those with a T3 or T4 tumor (all *p* values > 0.05). However, NPC patients with a N0 tumor had a higher level of 1 lipid whereas a lower level of another 112 lipids, compared with patients with a N1, N2, or N3 tumor (Table 6). NPC patients with a M0 tumor had a higher level of four lipids, whereas a lower level of another four lipids, compared with patients of a M1 tumor (Table 7). Finally, there was a lower level of 93 lipids, whereas a higher level of 1 lipid, among NPC patients with an early stage (stage II) cancer, compared with patients with an advanced stage (stage III, IVA or IVB) cancer (Table 8).

**Table 6:** Top 20 lipids with different levels between NPC patients with a N0 tumor and NPC patients with N1, N2, or N3 tumor.

No.	Lipid	Class	*p* Value^a^	FC^b^	Log_2_(FC)
1	TAG51:3-FA18:3	Glycerolipids	9.00E-09	0.146	−2.772
2	TAG47:0-FA14:0	Glycerolipids	5.49E-05	0.150	−2.735
3	TAG44:2-FA14:0	Glycerolipids	7.05E-05	0.158	−2.661
4	TAG46:3-FA18:3	Glycerolipids	1.95E-05	0.174	−2.525
5	TAG44:0-FA14:0	Glycerolipids	0.000989	0.174	−2.524
6	TAG44:0-FA16:0	Glycerolipids	0.000682	0.176	−2.503
8	TAG50:5-FA20:5	Glycerolipids	2.06E-05	0.183	−2.451
9	TAG42:0-FA16:0	Glycerolipids	0.00185	0.187	−2.417
10	TAG42:0-FA14:0	Glycerolipids	0.00186	0.188	−2.412
11	TAG46:0-FA18:0	Glycerolipids	7.73E-05	0.189	−2.401
12	TAG40:0-FA16:0	Glycerolipids	0.00262	0.208	−2.262
13	TAG50:4-FA20:4	Glycerolipids	7.23E-05	0.209	−2.259
14	TAG45:0-FA16:0	Glycerolipids	0.00022	0.209	−2.258
15	TAG45:0-FA14:0	Glycerolipids	0.00029	0.211	−2.245
16	TAG42:1-FA16:1	Glycerolipids	0.00256	0.212	−2.239
17	TAG42:1-FA14:0	Glycerolipids	0.00130	0.216	−2.211
18	TAG44:1-FA14:0	Glycerolipids	0.00071	0.220	−2.182
19	DAG14:0/18:3	Glycerolipids	0.00611	0.225	−2.151
20	PA18:1/22:5	Glycerophospholipids	0.01645	2.106	1.075

a *p* value was calculated using unpaired *t* test.

b Fold-change (FC) value was calculated by comparing the averages of NPC patients with a N0 tumor to that of patients with N1, N2, or N3 tumor.

**Table 7:** Lipids with different levels between NPC patients with a M0 stage tumor and NPC patients with a M1 stage tumor.

No.	Lipid	Class	*p* Value^a^	FC^b^	Log_2_(FC)
1	TAG40:0-FA14:0	Glycerolipids	0.0303	2.882	1.527
2	DAG16:0/20:3	Glycerolipids	0.0205	2.641	1.401
3	DAG18:1/20:1	Glycerolipids	0.0001	2.410	1.269
4	DAG16:1/20:0	Glycerolipids	0.0028	2.262	1.177
5	PG14:0/20:3	Glycerophospholipids	0.0247	0.476	−1.071
6	PEO-16:0/20:3	Glycerophospholipids	0.0062	0.455	−1.135
7	PS18:1/20:1	Glycerophospholipids	0.0184	0.443	−1.175
8	PS18:2/18:3	Glycerophospholipids	0.0001	0.439	−1.187

a *p* value was calculated using unpaired *t* test.

b Fold-change (FC) value was calculated by comparing the averages of NPC patients with a M0 tumor to that of patients with M1 tumor.

**Table 8:** Top 30 lipids with different levels between NPC patients with a stage II tumor and NPC patients with a stage III or IV tumor.

No.	Lipid	Class	*p* Value^a^	FC^b^	Log_2_(FC)
1	TAG40:0-FA14:0	Glycerolipids	0.003249	0.194	−2.368
2	LPE16:1	Lyso-Glycerophospholipids	9.34E-05	0.243	−2.042
3	LPE18:3	Lyso-Glycerophospholipids	0.000374	0.244	−2.037
4	TAG40:0-FA16:0	Glycerolipids	0.005182	0.258	−1.952
5	TAG44:0-FA16:0	Glycerolipids	0.003378	0.259	−1.948
6	TAG44:0-FA14:0	Glycerolipids	0.004962	0.264	−1.921
7	TAG44:2-FA14:0	Glycerolipids	0.000401	0.268	−1.901
8	TAG48:4-FA20:4	Glycerolipids	0.001858	0.269	−1.893
9	LPE22:4	Lyso-Glycerophospholipids	1.09E-05	0.273	−1.871
10	LPE20:3	Lyso-Glycerophospholipids	1.24E-05	0.281	−1.831
11	TAG44:1-FA14:0	Glycerolipids	0.001772	0.282	−1.826
12	LPE16:0	Lyso-Glycerophospholipids	9.76E-06	0.287	−1.800
13	TAG42:0-FA14:0	Glycerolipids	0.00826	0.296	−1.757
14	TAG42:1-FA16:1	Glycerolipids	0.008864	0.300	−1.738
15	LPE20:4	Lyso-Glycerophospholipids	3.43E-05	0.304	−1.719
16	TAG42:1-FA14:0	Glycerolipids	0.006886	0.307	−1.706
17	TAG45:0-FA14:0	Glycerolipids	0.003496	0.312	−1.682
18	TAG42:1-FA16:0	Glycerolipids	0.009011	0.312	−1.681
19	TAG50:5-FA20:5	Glycerolipids	0.000911	0.315	−1.667
20	LPE18:1	Lyso-Glycerophospholipids	0.000936	0.321	−1.639
21	LPE18:2	Lyso-Glycerophospholipids	0.000397	0.325	−1.621
22	LPE20:1	Lyso-Glycerophospholipids	0.000225	0.328	−1.606
23	LPE20:2	Lyso-Glycerophospholipids	0.000681	0.332	−1.591
24	TAG42:0-FA16:0	Glycerolipids	0.015866	0.337	−1.567
25	TAG47:2-FA18:2	Glycerolipids	0.000406	0.340	−1.558
26	TAG46:0-FA18:0	Glycerolipids	0.004683	0.348	−1.521
27	LPE18:0	Lyso-Glycerophospholipids	0.00014	0.349	−1.520
28	TAG44:1-FA16:1	Glycerolipids	0.012062	0.351	−1.513
29	LPE22:6	Lyso-Glycerophospholipids	0.000241	0.365	−1.454
30	PI^c^18:0/20:1	Glycerophospholipids	0.011228	2.164	1.114

a *p* value was calculated using unpaired *t* test.

b Fold-change (FC) value was calculated by comparing the averages of NPC patients with a stage II tumor to that of patients with stage III or IV tumor.

### Lipid Profiles in NPC Patients and Controls by Sex

Male NPC patients had a lower level of 8 lipids, whereas a higher level of 1 lipid, when compared to female NPC patients (Table 9). Statistically significant differences were also noted for 49 lipids between male and female controls (Table 10). Male controls had a lower level of 42 glycerophospholipids, whereas a higher level of seven free fatty acids, compared with female controls.

**Table 9:** Lipids with different levels between male and female NPC patients.

No.	Lipid	Class	*p* Value^a^	FC^b^	Log_2_(FC)
1	FFA14:1	Free fatty acids	3.66E-10	0.381	−1.391
2	MAG22:4	Glycerolipids	0.03007	0.406	−1.302
3	PA16:0/14:0	Glycerophospholipids	0.00862	2.398	1.262
4	PA16:0/20:3	Glycerophospholipids	0.00139	0.396	−1.335
5	PA18:2/20:3	Glycerophospholipids	0.01691	0.196	−2.351
6	PA18:2/20:5	Glycerophospholipids	0.00226	0.362	−1.466

a *p* value was calculated using unpaired *t* test.

b Fold-change (FC) value was calculated by comparing the averages of male NPC patients to that of female NPC patients.

**Table 10:** Lipids with different levels between male and female controls.

No.	Lipid	Class	*p* Value^a^	*FC* ^b^	Log_2_(FC)
1	FFA14:1	Free fatty acids	1.64554E-13	3.967	1.988
2	FFA18:0	Free fatty acids	1.35231E-05	2.774	1.472
3	FFA18:1	Free fatty acids	0.000137	2.447	1.291
4	FFA20:0	Free fatty acids	0.015721	2.129	1.090
5	FFA22:4	Free fatty acids	1.36018E-05	2.432	1.282
6	FFA22:6	Free fatty acids	0.026542	2.013	1.009
7	FFA24:0	Free fatty acids	1.56796E-11	2.320	1.214
8	PA16:0/14:0	Glycerophospholipids	0.012941	0.297	−1.752
9	PA18:1/16:1	Glycerophospholipids	0.013245	0.472	−1.082
10	PA18:1/20:5	Glycerophospholipids	0.017937	0.478	−1.064
11	PA18:2/20:5	Glycerophospholipids	0.028257	0.487	−1.039
12	PA20:0/22:4	Glycerophospholipids	0.000241	0.445	−1.169
13	PG14:0/20:3	Glycerophospholipids	0.000889	0.392	−1.351
14	PG14:0/22:4	Glycerophospholipids	0.000206	0.497	−1.010
15	PG14:0/22:6	Glycerophospholipids	8.20036E-06	0.440	−1.183
16	PG16:0/14:0	Glycerophospholipids	0.008946	0.455	−1.136
17	PG16:0/22:4	Glycerophospholipids	0.000519	0.490	−1.029
18	PG16:0/22:5	Glycerophospholipids	0.000196	0.474	−1.076
19	PG18:0/20:4	Glycerophospholipids	0.000279	0.494	−1.017
20	PG18:0/20:5	Glycerophospholipids	0.001311	0.461	−1.118
21	PG18:0/22:4	Glycerophospholipids	0.025707	0.464	−1.109
22	PG18:0/22:5	Glycerophospholipids	0.001247	0.415	−1.268
23	PG18:1/20:1	Glycerophospholipids	1.44853E-06	0.341	−1.551
24	PG18:1/22:4	Glycerophospholipids	0.001569	0.371	−1.430
25	PG18:1/22:5	Glycerophospholipids	2.51331E-06	0.430	−1.218
26	PG18:1/22:6	Glycerophospholipids	1.63772E-06	0.494	−1.018
27	PG18:2/18:2	Glycerophospholipids	4.21405E-05	0.499	−1.004
28	PG18:2/18:3	Glycerophospholipids	0.005372	0.498	−1.007
29	PG18:2/22:4	Glycerophospholipids	0.003607	0.369	−1.439
30	PG18:2/22:5	Glycerophospholipids	0.001102	0.424	−1.239
31	PG18:2/22:6	Glycerophospholipids	0.000178	0.461	−1.116
32	PG20:0/20:3	Glycerophospholipids	0.002385	0.395	−1.340
33	PI14:0/20:1	Glycerophospholipids	0.014398	0.459	−1.125
34	PI16:0/18:2	Glycerophospholipids	0.000819	2.024	1.017
35	PI18:1/20:1	Glycerophospholipids	0.009336	0.437	−1.196
36	PI18:1/22:4	Glycerophospholipids	0.003857	0.497	−1.008
37	PI20:0/16:1	Glycerophospholipids	7.93104E-05	0.489	−1.031
38	PS14:0/22:4	Glycerophospholipids	0.001487	0.485	−1.043
39	PS18:0/14:0	Glycerophospholipids	0.000922	0.362	−1.465
40	PS18:0/16:1	Glycerophospholipids	0.001689	0.451	−1.150
41	PS18:0/18:0	Glycerophospholipids	0.000817	0.483	−1.051
42	PS18:0/18:1	Glycerophospholipids	0.000728	0.463	−1.112
43	PS18:0/20:2	Glycerophospholipids	0.000141	0.499	−1.004
44	PS18:2/20:4	Glycerophospholipids	2.39592E-05	0.471	−1.085
45	PS18:2/22:4	Glycerophospholipids	1.46628E-05	0.428	−1.224
46	PS18:2/22:5	Glycerophospholipids	0.000865	0.494	−1.019
47	PS18:2/22:6	Glycerophospholipids	1.25561E-06	0.428	−1.223
48	PS20:0/20:3	Glycerophospholipids	0.000938	0.494	−1.017
49	PS20:0/22:6	Glycerophospholipids	7.96259E-05	0.493	−1.020

a *p* value was calculated using unpaired *t* test.

b Fold-change (FC) value was calculated by comparing the averages of male in controls to that of female.

## Discussion

In a study of 100 patients with NPC and 100 age- and sex-matched healthy controls, we used non-targeted lipid metabolomics techniques based on LC–MS/MS to compare the plasma lipid profiles of NPC patients to that of healthy controls and to analyze lipid profiles of the NPC patients by EBV antibodies, tumor stage, and sex. We found that 21 lipids, including 10 glycerophospholipids, 3 glycerolipids, and 8 free fatty acids, demonstrated differential levels between patients and controls. Among these lipids, majority showed a lower level, whereas 2 glycerolipids showed a higher level, among NPC patients than controls. We also found the lipid profiles to differ by the titer and presence of different EBV antibodies, as well as by tumor stage and sex.

Although plasma lipidomics has rarely been studied in NPC, reprogramming of energy metabolism has been recognized as a hallmark (Hanahan and Weinberg, 2011) and alteration of lipid metabolism is known as a prominent metabolic feature (Beloribi-Djefaflia et al., 2016; Cheng et al., 2018; Snaebjornsson et al., 2020) in cancer. Further, increased uptake of exogenous lipids is known to promote cancer cell proliferation, growth, invasion, and migration (Snaebjornsson et al., 2020), whereas serum lipids are energy sources for tumor progression (Ward and Thompson, 2012; Beloribi-Djefaflia et al., 2016). Similarly, although the exact roles of different lipids on NPC are unknown, existing literature has suggested potential mechanisms linking together lipids to carcinogenesis in general. For instance, the mTORC2 signaling pathway promotes carcinogenesis whereas mTORC2 stimulates *de novo* lipid synthesis, especially synthesis of sphingolipids and glycerophospholipids (Guri et al., 2017). Glycerophospholipids, including phosphatic acids, phosphatidylglycerols, phosphatidylinositols, phosphatidylserines and lyso-phosphatic acids, are the most abundant lipids observed in our study. Phosphatidylinositols are the precursor of the second messenger inositol 1,4,5-trisphosphate and diacylglycerol; the former controls cytosolic calcium levels and the latter activates the protein kinase C kinase family (Goncalves et al., 2018). An increase in phosphatidylinositols has been shown in renal clear cell carcinoma (Neumann et al., 2020) and colon cancer (Hofmanova et al., 2020). On the other hand, inhibition of lyso-phosphatic acids was shown to reduce tumor formation in mouse ovaries (Carrasco and Merida, 2007). Phospholipids are the most prominent membrane lipids in mammalian cells (van Meer and Sprong, 2004) and have been shown to be increased in tumor cells (Hofmanova et al., 2020), suggesting that elevated phospholipids may be essential for tumor cells to assemble membrane structure to sustain proliferation. In addition to phospholipids, intracellular lipids, especially fatty acids, and cholesterols, are also involved in tumor cell proliferation, apoptosis, stemness, invasiveness, and epithelial-mesenchymal transition (Koundouros and Poulogiannis, 2020). In a previous study, we found that fatty acids were upregulated whereas the ISG15-conjugating enzyme UbcH8 was epigenetically inactivated in NPC cells (Zhou et al., 2015). In another study, we further showed that the re-expression of *SLC27A6* might increase fatty acid uptake in NPC cells (Zhong et al., 2021).

EBV infection plays an important role in the development of NPC, especially in the endemic areas such as Southeast Asia. The titers of serum EBV antibodies are known to increase dramatically in NPC patients (Chen et al., 2015). Interestingly, EBV has also been shown to be able to infect primary human adipocytes *in vitro*, leading to increased release of free fatty acids, glycerol, and expression of proinflammatory adipokines (Liu et al., 2021). Further, EBV-encoded latent membrane protein 1 and EBV-encoded RNAs were shown to promote cell proliferation by upregulating fatty acid synthase in NPC cells (Lo et al., 2018) whereas EBV-encoded latent membrane protein 2A (LMP2A) were shown to promote lipid accumulation by blocking adipose triglyceride lipase in NPC cells, resulting in an enhanced cell migration (Zheng et al., 2020). Taken together, our results of different lipid profiles between NPC patients with different status of EBV antibodies suggest that EBV infection might modulate the risk and prognosis of NPC through the reprogramming of lipid metabolism.

In the host, EBV can establish two alternative modes of life cycle, known as latent infection and lytic infection. The switch from latent infection to lytic infection is known as EBV reactivation (Li et al., 2016). EBV found in NPC cells is mostly in latent cycle, with the viral genome existing as an episome in the nucleus and only a few viral genes, including EBNA1, expressed (Kempkes and Robertson, 2015). However, it is speculated that it is a small number of cells with lytic infection of EBV that promote NPC carcinogenesis and progression, because antibodies against lytic infection, such as EA-IgA and VCA-IgA, were found to be elevated several years before clinical onset of NPC, whereas levels of these antibodies were correlated with disease progression (Chien et al., 2001; Cao et al., 2011). It is therefore highly plausible that reactivation of EBV is critical in the initiation and progression of NPC. EBV antibodies are effective markers for different phases of EBV infection. In the present study, we found that NPC patients with positive EBNA1-IgA antibodies had a higher level of 143 lipids, but a lower level of only two lipids, compared to NPC patients with negative EBNA1-IgA antibodies. However, NPC patients with a higher titer of either EA-IgA or VCA-IgA antibodies had a lower level of lipids, compared to NPC patients with a lower titer of these antibodies. The different characteristics of lipid metabolism during the latent (indicated by positive EBNA1-IgA) and lytic (indicated by high titers of EA-IgA and VCA-IgA) phases of EBV infection suggest that altered lipid metabolism in relation to or as a result of EBV reactivation might be an important mechanism linking together EBV infection, especially EBV reactivation, to the development of NPC. When studying lipid profiles in relation to tumor stage of the NPC patients, we found that there was a lower level of many lipids but a higher level of only few lipids in early stage (e.g., N0 and stage II), compared with advanced stage (e.g., N1 and stage III, IVA or IVB) NPC, suggesting a dynamic change of lipid metabolism during tumor progression. It is interesting to note that these lipids are not the same as the lipids showing a different level between NPC patients and healthy controls. These results might therefore suggest that different characteristics of lipid metabolism are present in the initiation and progression of NPC. Further, NPC patients with advanced stage tumor demonstrated only a lower level of 1 lipid, namely phosphatidylinositol 18:0/20:1, compared with NPC patients with early-stage tumor. In the comparison between NPC patients and healthy controls, we also found that NPC patients demonstrated a lower level of many phosphatidylinositols. It is therefore possible that these phosphatidylinositols are originated from NPC cells and are regulated during NPC progression. If this hypothesis is true, phosphatidylinositols might be of potential use as biomarkers for the diagnosis and prognostic indication of NPC. However, as the pattern was less clear in terms of T stage and metastasis status, these findings need to be validated in future studies.

The sex-specific results among NPC patients and healthy controls suggest distinct lipid profiles between male and female, which are independent of NPC status. Although further research is clearly needed in unraveling the underlying mechanisms for such pattern, we speculate that sex-specific factors such as sex hormones might contribute.

The main strengths of the study are the use of lipidomics analysis based on LC–MS/MS and the rich data on clinical characteristics for NPC patients such as EBV antibodies and TNM and clinical stages. Given the cross-sectional design of the present study, a temporal relationship cannot be examined between EBV infection, alterations in lipid profile, and onset of NPC, precluding the possibility to discuss causation. The observed lipid profile alterations among patients with NPC, compared with healthy controls, might therefore be attributable to different reasons. First, the alterations might be due to epiphenomena, i.e., the lipids change as a result of NPC. Second, the alterations are secondary to EBV infection, especially EBV reactivation, which leads subsequently to the development of NPC. Third, the alterations are causally related to NPC development, i.e., changed lipid profiles, independently of or jointly with EBV infection, lead to the development of NPC. Further, as our primary aim was to understand the potential role of altered lipid metabolism in the causation of NPC, we only analyzed lipid profiles before any treatment was given, precluding the possibility to examine the role of cancer treatment on lipids. Prospective studies with longitudinal plasma samples collected from long before onset of NPC (preferably at the time of EBV reactivation) until after cancer diagnosis (preferably after cancer treatment) will help to better understand the sequence of these events as well as the impact of cancer treatment on lipid profiles.

Another limitation of the study is the lack of separate assay to validate the observed lipid profiles using LC–MS/MS. This is however not unique to the present study as such validation is currently non-existent in lipidomics studies. A further limitation of the study is the lack of independent samples to validate the observed alterations in lipid profiles among patients with NPC, compared to healthy controls. As a result, we deemed the present study as a pilot effort and call for future studies to confirm (or refute) our findings. We did not focus on the prediction ability of individual lipids in the study, but rather the prediction ability of a composite lipid profile. The prediction ability of the model is likely influenced by sample size as well as noise due to confounding factors and the accuracy of lipid measurements. Future studies with larger sample size and better control of confounding are therefore needed to further assess the prediction ability of our model. Finally, as our study was based on an endemic area of NPC, whether our findings are generalizable to NPC in non-endemic areas remains to be examined.

## Conclusion

In conclusion, plasma lipidomics might help to differentiate NPC cases from controls whereas EBV infection might contribute to the risk and prognosis of NPC through modulating lipid metabolism in tumor cells as well as in the periphery.

## Data Availability Statement

The raw data supporting the conclusions of this article will be made available by the authors, without undue reservation.

## Ethics Statement

The studies involving human participants were reviewed and approved by ethics approval of this study was obtained from the Ethical Evaluation Committee of Wuzhou Red Cross Hospital (No. LL2017-19, Wuzhou, China). Written informed consent to participate in this study was provided by the participants’ legal guardian/next of kin.

## Author Contributions

ZZ and YC conceived the idea and designed the study. YH and JL performed the experiments. WH, XP, YL, XZ, and YY contributed to the data analysis. XX and WZ drafted the manuscript. XZ contributed to the result interpretation, critically reviewed the manuscript, and supervised experiments. All authors contributed to the article and approved the submitted version.

## Funding

This work was supported by the National Natural Science Foundation of China (81960490, 81760489, 82060511, and 81860601), the Guangxi Natural Science Foundation of China (2020GXNSFAA297105), the Youth Program of Guangxi Natural Science Foundation of China (2018GXNSFBA281158, 2018GXNSFBA281028), the High-level Talent Introduction Plan of the First Affiliated Hospital of Guangxi Medical University (the fifth level), and the fund of Guangxi Key Laboratory of Early Prevention in Regional High Incidence Cancer (GKE-ZZ 202119).

## Conflict of Interest

The authors declare that the research was conducted in the absence of any commercial or financial relationships that could be construed as a potential conflict of interest.

## Publisher’s Note

All claims expressed in this article are solely those of the authors and do not necessarily represent those of their affiliated organizations, or those of the publisher, the editors and the reviewers. Any product that may be evaluated in this article, or claim that may be made by its manufacturer, is not guaranteed or endorsed by the publisher.

## Abbreviations

NPC: Nasopharyngeal Carcinoma
EBV: Epstein–Barr virus
EA: Early Antigen
VCA: Viral Capsid Antigen
EBNA1: EBV Nuclear Antigen 1
Zta-IgA: BZLF1 Transcription Activator Protein
PLS-DA: Partial Least Squares Discriminant Analysis
OPLS-DA: Orthogonal Partial Least Squares Discriminant Analysis
LC–MS/MS: Liquid Chromatography-Tandem Mass Spectrometry
